# SNAP23 promotes the malignant process of ovarian cancer

**DOI:** 10.1186/s13048-016-0289-9

**Published:** 2016-11-17

**Authors:** Qi Sun, Xing Huang, Quanli Zhang, Junwei Qu, Yang Shen, Xin Wang, Haijun Sun, Jie Wang, Lin Xu, Xiaoxiang Chen, Binhui Ren

**Affiliations:** 1 Department of Jiangsu Key Laboratory of Molecular and Translational Cancer Research, Cancer Institute of Jiangsu Province, Nanjing, Jiangsu China 210009; 2 Department of Cardiothoracic Surgery, Jinling Hospital, Southern Medical University, East Zhongshan Road 305, Xuanwu District, Nanjing, Jiangsu 210002 People’s Republic of China; 3 Department of Pathology, Jiangsu Cancer Hospital, Baiziting 42, Nanjing, 210009 People’s Republic of China; 4 Department of Gynecologic oncology, Jiangsu Cancer Hospital, Baiziting 42, Nanjing, 210009 People’s Republic of China; 5 Department of Thoracic Surgery, Jiangsu Cancer Hospital, Baiziting 42, Nanjing, 210009 People’s Republic of China

**Keywords:** SNAP23, SNARE, Ovarian cancer, Apoptosis

## Abstract

**Background:**

Ovarian cancer (OC) was the primary malignant gynecological cancer and SNARE protein is closely related with tumor progression. Here, we identified SNAP23, a member of SNARE complex, as a potential oncogene in OC.

**Methods:**

We determined the expression of SNAP23 in OC tissues and explored the clinical significance through bioinformatics analysis. The effects of SNAP23 on OC cell proliferation, migration, invasion, cell cycle and apoptosis were then evaluated in vitro.

**Results:**

SNAP23 is hyper-expressed in OC tumor tissues compared to normal tissues, and increased expression of SNAP23 is associated with a poor progression free survival (HR = 1.24, 95% CI = 1.07–1.44, *p* = 0.0042). SNAP23 knock down increases cell apoptosis and inhibits cell proliferation, migration and invasion of OC cells. GO analysis reveals that most genes correlated highly with SNAP23 were enriched in metabolic process.

**Conclusions:**

Our data suggest that SNAP23 may serve as an oncogene promoting tumorigenicity of OC cells by decreasing apoptotic process.

## Background

Ovarian cancer (OC) is the primary malignant gynecological cancer and accounts for the fifth leading cancer in women, mostly found in advance stage. Due to the lack of effective methods for advance cases, overall survival of OC is poor [1–4]. Therefore, identifying tumor genesis mechanisms for OC is urgent.

Cellular organelles in eukaryotic cells as well as tumor cells mainly transport materialsvia vesicles which is dependent on SNAREs [soluble NSF(N-ethylmaleimide-sensitive fusion protein) attachment protein receptors] [5, 6]. There is some evidence that SNAREs contribute to tumor invasion and metastasis through trafficking of matrix metalloproteinases (MMPs) [7, 8]. VAMP (Vesicle-associated membrane protein), SNAP-25 (Synaptosome associated protein of 25 kDa) and Syntaxin constitute the basic SNARE complex. SNAP-25 family genes, include four homologues, SNAP23, SNAP25, SNAP29 and SNAP47, in human [9, 10]. There is recent evidence that a regulated membrane trafficking pathway mediated by SNAP23 is required for breast cancer cell invadopodium formation and efficient tumor cell invasion in vitro. We recently identified SNAP47 as a potential novel oncogene in NSCLC [11] and SNAP23 mediated the vesicle transport and efflux secretion of normal airway epithelium [12]. Seeking ovarian cancer related SNAP proteins may help to reveal new mechanisms of invasion and metastasis of ovarian cancer, and provide new ideas for clinical diagnosis and treatment.

In the present study, SNAP23 was found to be a potential oncogene that promotes OC cell proliferation and migration in vitro. Moreover, apoptotic pathway blockage and metabolic abnormalities may contribute to the oncogenic function of SNAP23. SNAP23 is required for efficient invasion by OC cancer cells. The impaired proliferation and reduced invasive capacity observed in cells as a consequence of inhibiting SNAP23 function suggests an important role for SNARE mediated pathway in OC progression.

## Methods

### Bioinformatics analysis

To explore the expression of SNAP23 protein in ovary normal tissues and OC tissues, we used Human Protein Atlas (http://www.proteinatlas.org) to evaluate the expression level following the guidance [13–16]. For exploring potential molecular mechanism, we analyzed the data of TCGA by using the cBioPortal [17](http://www.cbioportal.org/) and seeking for genes with high expression correlation with SNAP23 with the help of the co-expression and enrichment analysis tool. Then the gene list was submitted to DAVID Bioinformatics Resources 6.7 (http://david.abcc.ncifcrf.gov) for KEGG pathway enrichment analysis [18, 19]. KM-plotter (http://kmplot.com/) was used to explore the influence of SNAP23 expression on survival of OC patients.

### Cell lines and cell culture

Human ovarian cancer cell lines A2780, SK, HO8910, HO8910PM and human ovary epithelium cell line HOSE were purchased from Shanghai Institutes for Biological Science, China. All cell were grown in RPMI 1640 medium (Kaiji, Nanjing China), supplemented with 10% fetal bovine serum (FBS, Gibico) and penicillin/streptomycin and cultured at 37 °C in a humidified incubator containing 5% CO_2._

### RNA extraction, reverse transcription and real-time quantitative PCR

OC cells were harvested and collected before extraction. Total RNA was extracted with Trizol reagent (Invitrogen, Carlsbad, CA, USA) according to the manufacturer’s protocol. 1.0ug total RNA was reversely transcribed in a final volume of 20ul cDNA using the PrimeScript RT Master Mix (Takara, Cat. #RR036a). The quantitative real-time polymerase chain reaction (qRT-PCR) was performed with the SYBR Select Master Mix (Applied Biosystems, Cat: 4472908). The qRT-PCR was performed on QuantStudioTM 6 Flex Real-Time PCR System and the qRT-PCR reaction was run under the following steps: 95 °C for 10 min, followed by 40 cycles of 92 °C for 15 sec and 60 °C for 1 min. Each sample was run in triplicate and the relative expression was calculated and normalized using the 2^-ΔΔCt^ method relative to actin.

### siRNA transfection

SK and A2780 cell lines were placed into 6-well plate and cultured for 24 h before transfection. Small interface RNA with specific sequences targeting SNAP23 or negative control (RealGene, Nanjing, China) were transfected into OC cells with Lipofectamine RNAi max reagent (Invitrogen, USA) according to the manufacturer’s introduction. Both q-PCR and western blot were used to evaluate the transfection efficiency. Two siRNAs were designed, and the sequences were as follows: siRNA1 for SNAP23: sense, 5’- GAGUCUGGCAAGGCUUAUATT -3’ and antisense, 5’- UAUAAGCCUUGCCAGACUCTT -3’; siRNA2 for SNAP23: sense, 5’- GCUUGGACCAAAUAAAUAATT -3’ and antisense, 5’- UUAUUUAUUUGGUCCAAGCTT -3’. The following non-sense siRNA was used as the control: sense, 5’-UUCUCCGAACGUGUCACGUTT-3’ and antisense, 5’- ACGUGACACGUUCGGAGAATT -3’.

### Protein extraction and western blot

Transfected cells were harvested and protein was extracted using the RIPA Lysis Buffer (Kaiji, Nanjing, China) followed the protocol provided by the manufacture. The quantitative of protein was measured by the BCA Protein Assay Kit (Kaiji, Nanjing, China). Then equal amount of proteins were loaded in the SDS page gel and transferred onto a PVDF membrane after the electrophoresis. After blocked with defatted milk for 2 h, the membrane was incubated all night with antibodies against SNAP23 (Abcam, ab42483 1:1000) or GAPDH (CST,2118, 1:1000). After washing with TBST 3 times, the membrane was incubated with goat anti-rabbit HRP-conjugated secondary antibody (1:10,000; Abcam) or goat anti-mouse HRP-conjugated secondary antibody (1:10,000; Abcam) for 2 h at room temperature. The bands were visualized by ECL detection (Thermo Scientific), and all the experiments were repeated triple times.

### Clonogenic assay

A 2 ml complete medium with 200 cells (transfected with si-SNAP23 or NC) was added into 6-well plate. The plate was put in the carbon dioxide cell incubator with humidified air at 37 °C and 5% CO_2_, replacing medium every 3 or 4 days. After 2 weeks, medium was removed and cells were fixed with 4% paraformaldehyde for 30 min and stained with 0.1% crystal violet for 1 h. Visual colons were manually counted and each experiment was repeated 3 times.

### Cell proliferation assay

The cell proliferation rate was measured using a Cell Counting Kit-8 (Kaiji, Nanjing, China). Cells were plated in a 96-well plates at a destiny of 2000 cells in 100ul per well and cultured at 37 °C and 5% CO_2._ After 6 h, cells were added with 10ul CCK-8 solution according to manufacturer’s protocol and the OD value at 450 nm was measured with microplate reader. Each experiment was repeated quadruplicate at various time points for 6 days.

### Cell migration, invasion and wound healing assays

For migration assays, transfected cells (40,000 cells in 100ul per well) were plated in the upper chamber of trans-well assay inserts (8 mm pores, Millipore, Billerica, MA) containing 200ul of serum-free RPMI1640 media. The lower chambers were filled with RPMI1640 containing 10% FBS. After 24 h of incubation, cells on the filter surface were fixed with methanol, stained with crystal violet, and photographed. Migration was assessed by counting the number of stained cell nuclei from 5 random fields per filter in each group.

For invasion assays, transfected cells (40,000 cells in 100ul per well) were plated in the top chamber with a matrigel-coated membrane (BD Biosciences) in 300ul serum-free RPMI1640. The bottom chambers were filled with RPMI1640 containing 10% FBS. Invasion was determined after 48 h incubation.

For wound healing assay, cells were seeded and transfected on six-well plates with si-SNAP23or si-NC, then an artificial scratch wound on a confluent monolayer of A2780 or SKcells was created with a 200-μl pipette tip. Serum-free medium was added for a further 24-h, and cells were imaged 24 h later. Each experiment was repeated three times.

### Flow-cytometry analysis

Transfected cells were cultured for 48 h and harvested for flow-cytometry analysis. After the double staining with fluorescein isothiocyanate (FITC)-Annexin V and propidium iodide was done by the FITC Annexin V Apoptosis Detection Kit (BD Biosciences) according to the manufacturer’s instruction. The cells were analyzed with a flow cytometry (FACScan; BD Biosciences) equipped with a Cell Quest software (BD Biosciences).

Cells for cell-cycle analysis were fixed with 75% alcohol and stored in −20 °C overnight and then stained with propidium oxide by the Cycle TEST PLUS DNA Reagent Kit (BD Biosciences) following the protocol and analyzed by FACScan. The percentage of the cells in G1, S, and G2–M phase were counted and compared.

### Statistical analysis

Data are presented as means ± S.D. and statistical analysis was performed using Student’s *t* test or one-way ANOVA (SPSS Statistics, version 20.0, Chicago, IL). *P* < 0.05 were considered statistically significant. The data graphs were made with GraphPad Prism 6.0 software.

## Results

### SNAP23 is hyper-expressed in OC tumor tissues

**Fig. 1:**
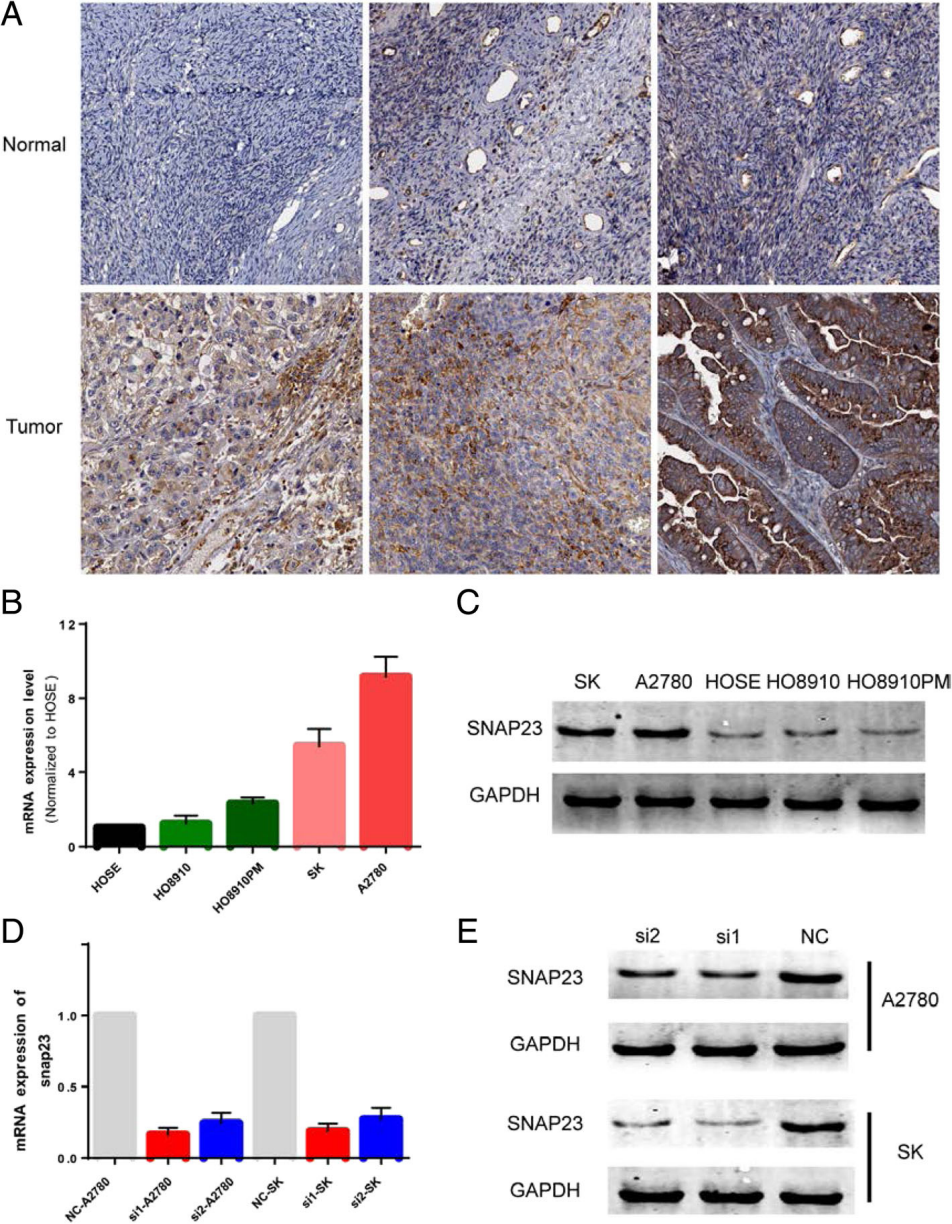
SNAP23 is highly expressed in OC tissues and cells. **a**. Immunohistochemistry analysis in The Human Protein Atlas revealed that normal ovarian tissues nearly express SNAP23, but most ovarian tumor tissues are positive for SNAP23. Protein was expressed at high levels in tumor tissues. **b** and **c**. The results of qRT-PCR and western blot assays showed that SNAP23 mRNA and protein are hyper-expressed in most ovarian cancer cell lines. **d** and **e**. Both of two designed siRNAs showed favorable inhibition, and siRNA1 had a better efficiency

By analyzing the Human Protein Atlas, we compared the protein expression of SNAP23, SNAP25, SNAP29 and SNAP47 in ovarian normal and tumor tissues. As a result, among the four SNAP proteins, SNAP23 showed the least expression in normal tissues and expressed abundantly in OC tissues. The Human Protein Atlas immunohistochemistry (IHC) analyses showed that SNAP23 was rarely expressed in normal ovary tissues, while was expressed in most ovarian cancer tissues (9 of 11) (Fig. 1a).

### Knockdown of SNAP23 inhibits the proliferation, migration and invasion of OC cell lines in vitro

The expression of SNAP23 was compared in different OC cell lines. SNAP23 was hyper-expressed in A2780 and SK cell lines as compared with human ovary epidermis (HOSE) cells by using quantitative PCR. As a result, SNAP23 was up-regulated in most OC cell lines compared with HOSE. And SNAP23 was significantly highly expressed in A2780 and SK cell lines (Fig. 1b). And the western blot analysis reported the same result (Fig. 1c).

**Fig. 2:**
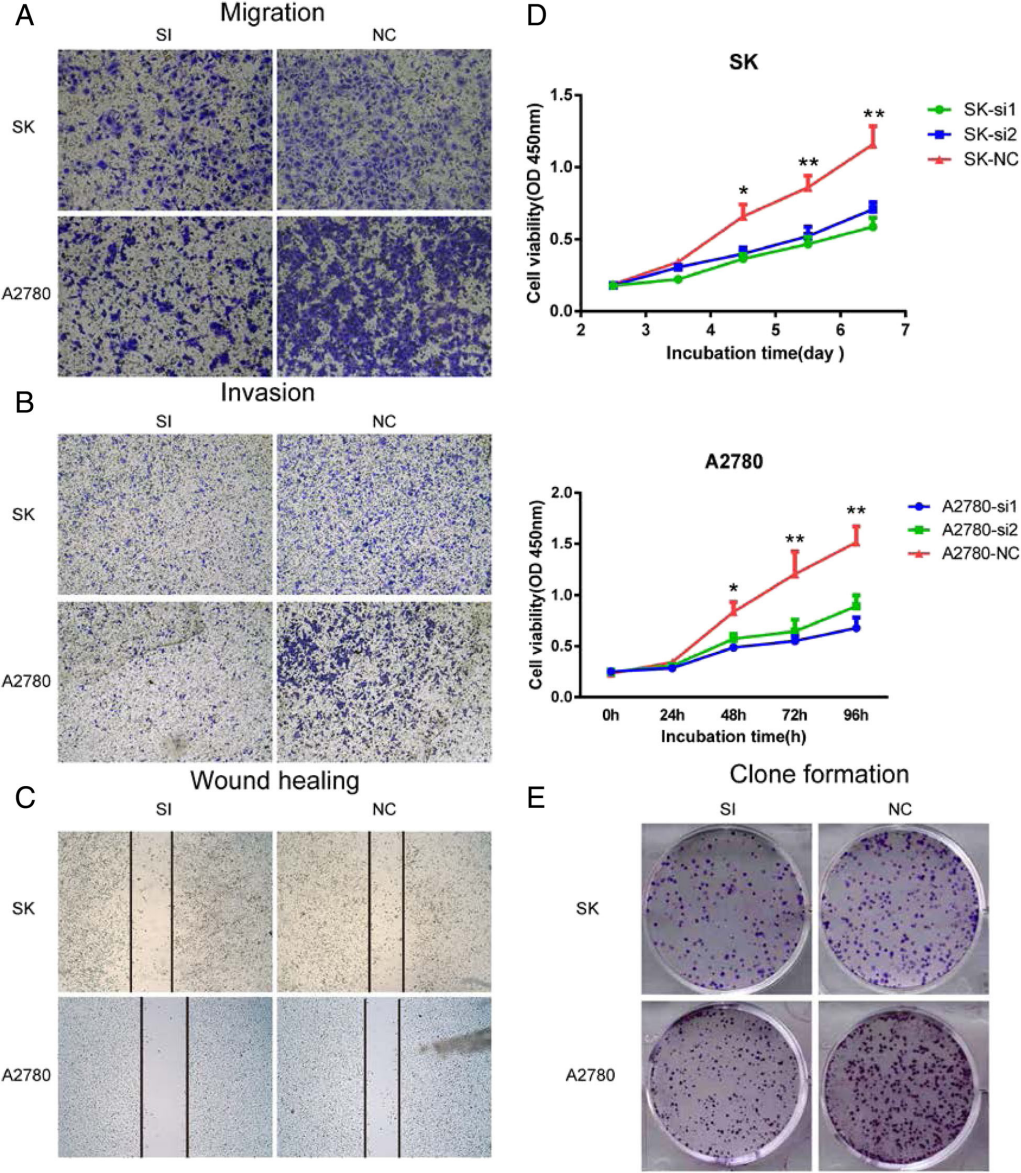
Knockdown of SNAP23 alters OC cell line proliferation, migration and invasion in vitro. **a**. Both of the designed siRNAs showed favorable inhibitory efficiency, and siRNA1 had a better efficiency than siRNA2. **b**. Depletion of SNAP23 undermined both SK and A2780 cells (*p* < 0.05). **c**. Colony numbers of SK and A2780 cells transfected with si-SNAP23 are less than those transfected with si-NC (*p* < 0.001). **d**. Migratory and invasion rates of SK and A2780 cells transfected with si-SNAP23 are decreased compared with NC group. **e**. Si-SNAP23 impaired migration as compared with NC group in wound healing assay (*p* < 0.001)

To investigate the biological function of SNAP23 in vitro, two small interface RNA targeting SNAP23 (siRNA-1 and siRNA-2) were designed and transfected into A2780 and SK cells to knock down the expression of SNAP23. Both siRNA constructs were able to effectively decrease SNAP23 mRNA and protein levels (Fig. 2a).

As shown in Fig. 2b, Cell-count-kit 8 analysis revealed that knockdown of SNAP23 significantly reduced the proliferation of A2780 and SK cells. Meanwhile, si-SNAP23 transfected cells had fewer colonies than those transfected with scramble siRNA (Fig. 2c).

The trans-well assay showed that migration of A2780 and SK cells were inhibited by siRNA-mediated knockdown of SNAP23 (Fig. 2d), and the wound healing assay yielded similar results (Fig. 2e). The matrigel invasion assay also revealed that si-SNAP23 treatment impaired the invasion capacities of A2780 and SK cells (Fig. 2d).

### Knockdown of SNAP23 increases OC cell apoptosis

**Fig. 3:**
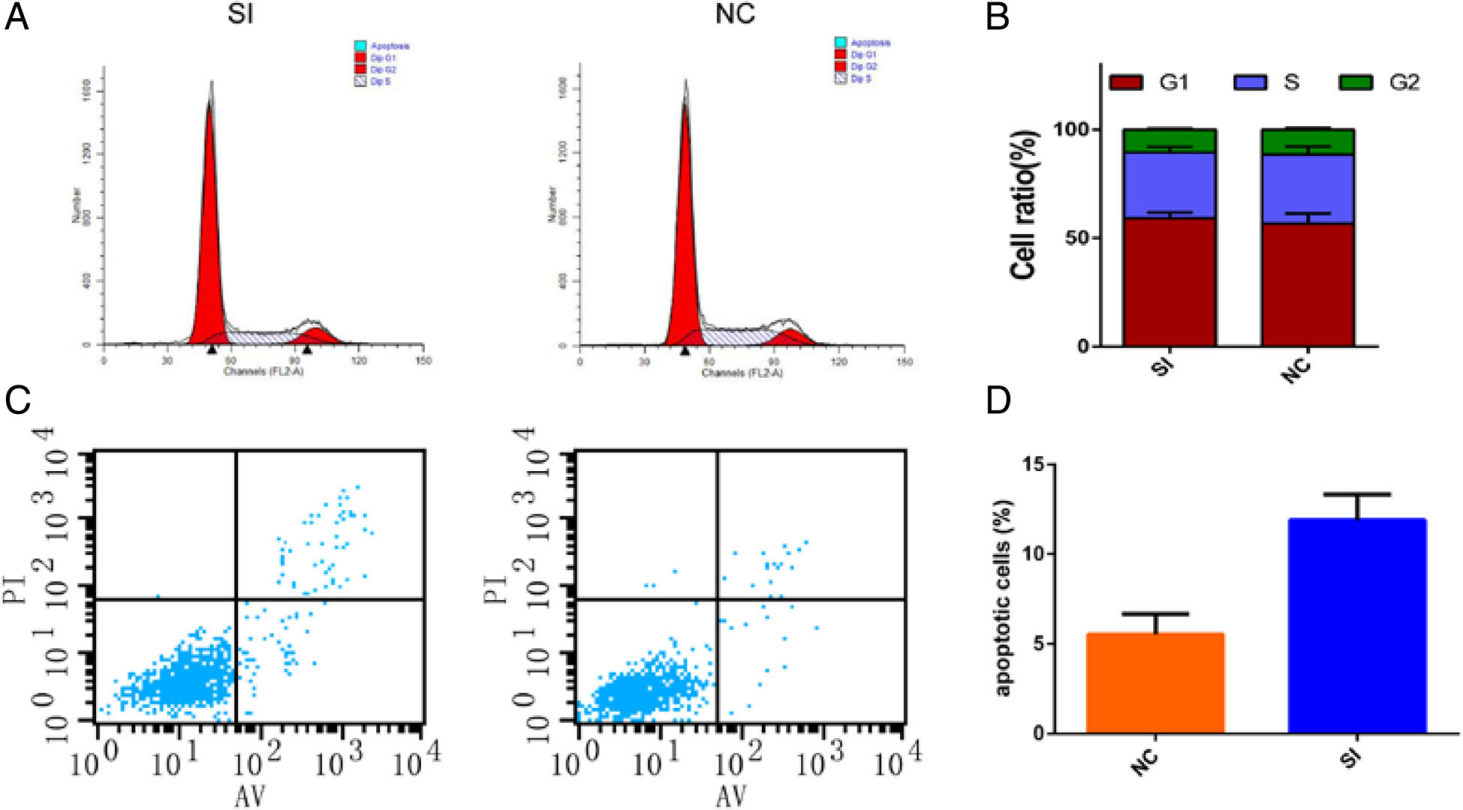
SNAP23 depletion increase OC cells apoptosis without influencing on cell cycle in vitro. **a** and **b**. SK cells transfected with si-SNAP23 led to apoptosis increase compared to si-NC. **c** and **d**. Silencing SNAP23 of SK cells showed no influence on cell cycle stage

We then performed flow cytometry to evaluate the influence of SNAP23 depletion on cell cycle change and apoptosis. As shown in Fig. 3a and b, si-SNAP23 treatment increased cell apoptosis compared with control group. After knocking down the expression of SNAP23,there was no significant change of cell cycle (Fig. 3c and d).

### Increased expression of SNAP23 is correlated with a poor Progression-Free-Survival and Bioinformatics analysis reveals SNAP23 is highly related with metabolic process

**Fig. 4:**
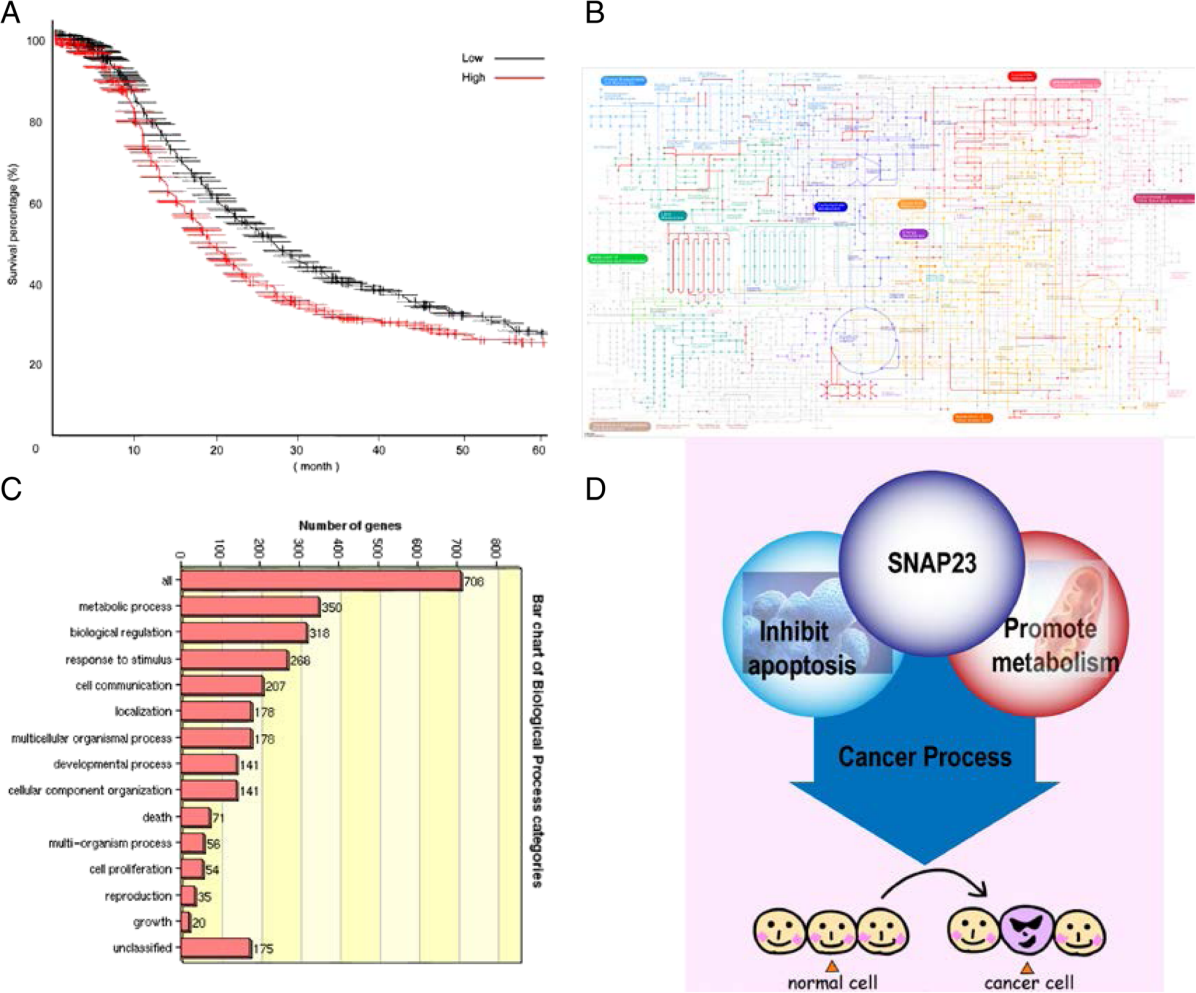
SNAP23 associated with a poor PFS and may influence apoptotic and metabolic processes in OC. **a**. KM-ploter analysis revealed that high expression of SNAP23 was associated with a poor progress-free-survival of OC patients (1187 patients were involved, and the median PFS of SNAP23 high expression were 17.0 months, while SNAP23 low expression 19.3 months). **b** and **c**. GO enrichment analysis indicated that SNAP23 expression was highly correlated with genes enriched in the metabolic process and biological regulation. **d**. We hypothesized that SNAP23 might influence cancer process via inhibiting apoptosis and promoting metabolic process

We used the website of Kaplan Meier-plotter to evaluate the influence of SNAP23 on OC patents survival. The result revealed that OC patients with highly expression of SNAP23 had a poor Progression-Free-Survival (HR = 1.23, *p* = 0.0023, Fig. 4a). We next used KEGG pathway and GO analysis (DAVID Bioinformatics Resources 6.7) on a list of genes co-expressed with SNAP23 that was obtained from cBioportal using both RNA-seq and microarray results of Ovarian Cancer (TCGA, Provisional). As shown in Fig. 4b, most of the genes co-expressed with SNAP23 were enriched in metabolic process and biological regulation. Given our findings that SNAP23 knockdown decreased cancer cell proliferation, migration, and invasion and inhibited apoptosis, we sought to hypothesize that SNAP23 might influence cancer progression via inhibiting apoptosis and promoting metabolic process (Fig. 4c).

## Discussion

SNAREs is a multifunctional protein family which was divided into V-SNARE on vesicles and T-SNARE on the plasma membrane according to subcellular localization. V-SNARE and T-SNARE form a complex and catalyze membrane fusion together [20–22]. Recent studies have identified that SNARE proteins are involved in several aspects of tumori-genesis. SNARE proteins could regulate cancer cell invasion as well as chemo-resistance.In addition, SNARE proteins are involved in cell development, transparent of autocrine and cell cycle progression [23–27]. Certain SNARE proteins were found to be closely associated with cancer progression.SANP23 is one of subclass proteins of T-SNARE expressed in many tissues and plays an important role in non-neuronal cells exocytosis [28]. SNAP23, also known as syndet, is considered as the paraplastic form of SNAP-25 other than nerve tissue [29]. SNAP23 was reported to be involved in secretion of matrix metalloproteinases, degradation of the extracellular matrix and cell invasion [30]. And our research was to explore whether SNAP23 could influence the ovarian cancer progression.

In this study, we present evidence that SNAP23 is over-expressed in OC tissues compared with normal ones. And SNAP23 is also hyper-expressed in several OC cell lines as compared to a normal HOSE cell line. CCK-8 assayand colony formation assay showed that knock down SNAP23 inhibited the proliferation and tumorigenesis of OC cells. Trans-well and Matrigel assays further revealed that silencing SNAP23 undermines cancer cell migration and invasion capacities. The flow cytometry analysis revealed that cell apoptosis increased in si-SNAP23 group, with no effects onthe cell cycle indicating that SNAP23 could influence tumorigenesis by inhibiting cell apoptosis, which was consistent with previous reports [31–33]. KM plot analysis suggests that ovarian cancer patients strongly expressing SNAP23 has a poor progression free survival than those with absent or weaker SNAP23 expression.

In order to seek further potential molecular mechanisms, we first identified and collected a list of highly co-expressed genes with SNAP23 through bioinformatics analysis. The GO analysis showed that SNAP23 was closely related with metabolic process, which may be a reason for cancer progression. SNAP23 has been reported to be related with lipid dropletfusion[34], and ablation of SNAP23 using siRNA reduced lipid droplets-mitochondria complex formation and beta oxidation[35].

Recent studies have found that there are not only gene abnormalities, but also metabolic abnormalities, especially energy metabolism abnormalities in the malignant tumor. In the 1920s, German biochemical and physiologist Warburg had observed that tumor cells could consume glucose and produce lactate under aerobic conditions (Warburg effect). Since the 1990s, with the use of fluorodeoxyglucose positron emission tomography (FDG-PET) technology, the tissue glucose intake could be detected by imaging, and Warberg effect was verified in a growing number of tumor types. In recent years, it is of great interest to explore strategies to inhibit the generation of energy in cancer cells by blocking energy metabolism-related pathways to achieve the treatment of malignant tumors. One of the hallmarks of cancer cells is the increased rate of adipogenesis. A recent study found that lipid-related genes such as SNAP23 were important for cell transformation and were significantly elevated in advanced breast and prostate cancers [36]. In the SNAP23-related gene analysis, the top 45 of 100 highest-ranking genes were closely related to metabolic processes, suggesting that SNAP23 as a key lipid metabolism-related genes and may play an important role in cancer cell metabolic process abnormalities. Generally speaking, SNAP23 might influence cancer process via promoting metabolic and inhibiting apoptosis.

## Conclusions

In conclusion, our study identified SNAP23 as a novel oncogene in Ovarian Cancer. These experiments show that SNAP23 is over-expressed in OC. SNAP23 could promote the proliferation, migration and invasion of OC in vitro, and is expected to become a new target for OC molecular diagnostics and therapy.

## Abbreviations

CCK8: Cell counting kit-8
NSCLC: Non-small cell lung cancer
q-PCR: Real-time Quantitative PCR Detecting System
siRNA: small interface RNA
TCGA: The cancer genome atlas.
